# Exudative pleurisy of coccidioidomycosis: A case report and review of the literature

**DOI:** 10.1186/1752-1947-2-291

**Published:** 2008-09-03

**Authors:** Kamyar Afshar, Ayana BoydKing, Om P Sharma

**Affiliations:** 1 Division of Pulmonary and Critical Care Medicine, Keck School of Medicine, University of Southern California, 1200 North State Street GH 11900, Los Angeles, CA 90033, USA

## Abstract

**Introduction:**

Community-acquired pneumonia is the most common manifestation in primary coccidioides infections (*Coccidioides immitis, C. posadasii*). It is essential that this endemic dimorphic fungus be considered in order to proceed with the most appropriate diagnostic tools and therapy.

**Case presentation:**

We present a rare case of primary pleural coccidioides and a review of the current literature for optimal diagnostic methods and therapeutic strategies.

**Conclusion:**

With increased domestic and international travel, coccidioidomycosis will likely be encountered in nonendemic regions. Recognition by physicians is critical for a timely diagnosis and therapy. Tissue culture can assist in the diagnosis and polymerase chain reaction analysis shows potential as a possible addition.

## Introduction

Coccidioides species, which are dimorphic fungi, are endemic to the Southwest United States and focal regions in Central and South America. With increased domestic and international travel, physicians must take a thorough travel history to consider coccidioides infection, given its non-specific presenting symptoms. Fortunately, infection with coccidioides does not always lead to clinical disease and may even result in lifelong cellular immunity. Typical clinical manifestations of this fungus include malaise, fever, cough and other non-specific symptoms that are indistinguishable from an influenza infection. We present a case of primary pleural coccidioidomycosis to add to the literature [1-3] and discuss the diagnostic tools that can assist in confirming the presence of a sole pleural effusion as a rare manifestation of this disease.

## Case presentation

A 39-year-old man was admitted in October 2006 with a 2-week history of sharp, non-radiating pain of the right shoulder blade with associated dyspnea upon exertion and 5 kg loss of weight. He denied fever, chills, night sweats or cough. His symptoms did not interfere with his occupation as a gardener. Vitals demonstrated a normotensive, afebrile 155 cm, 100 kg man with an oxygen saturation of 96% on room air.

**Figure 1:**
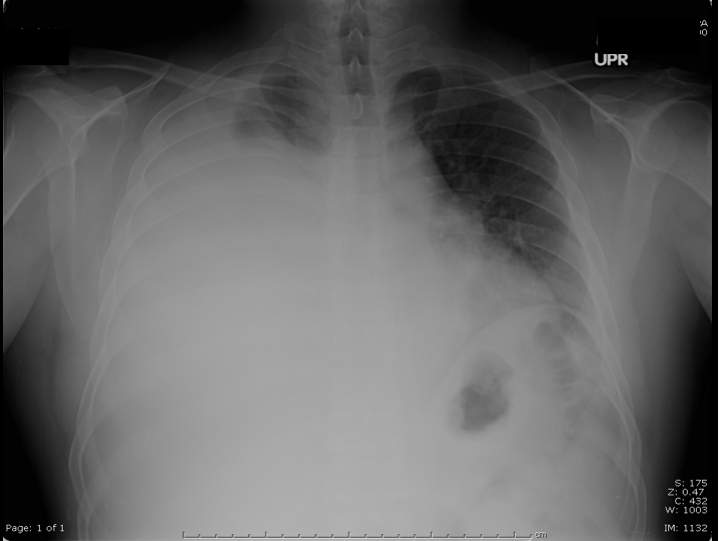
Chest radiograph showing moderate right pleural effusion.

**Figure 2:**
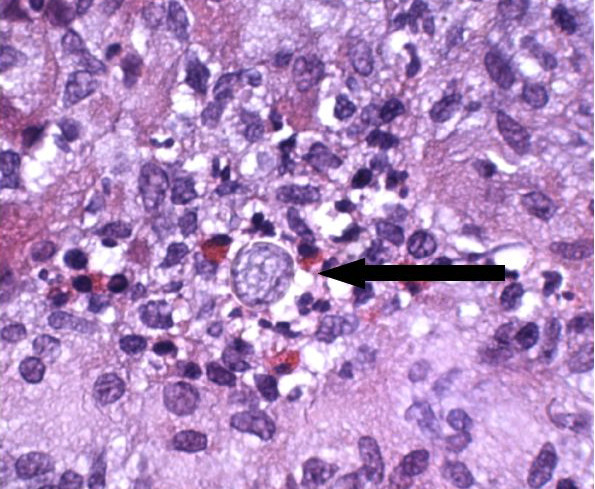
Closed pleural biopsy showing coccidioidomycosis with evidence of endospores.

Physical examination was normal with the exception of decreased breath sounds half way up the right lung field along with dullness to percussion and without tactile fremitus. A chest radiograph showed a moderately sized, right pleural effusion (Figure 1). The right thoracentesis fluid analysis showed a slightly cloudy and yellow fluid. Cell count results were 1,164 nucleated cells, 12% polymorphonuclear leukocytes, 80% lymphocytes, 7% monocytes, 1% eosinophils, glucose 123 mg/dl, lactate dehydrogenase (LDH) 103 units/l, and protein 5.3 g/dl (pleural to serum protein ratio, 0.8; pleural to serum LDH ratio, 0.7). Bacterial Gram stain and culture, acid-fast bacilli smears, fungal culture and cytology were all negative. Histopathological evaluation of the pleural biopsy noted granulomatous inflammation and fungal elements consistent with coccidioides (Figure 2). Cultures for tuberculosis remained negative even after 7 weeks. Purified protein-derivative skin test to the right forearm produced a 0 mm induration. Tests for human immunodeficiency virus were negative by both enzyme-linked immunosorbent assay and Western blot. Serum coccidioidomycosis complement fixation was normal (< 1:2). A post-thoracentesis chest radiograph did not reveal any evidence of parenchymal infiltrate. After several weeks of fluconazole therapy, the patient improved clinically, and follow-up chest radiograph showed near-complete resolution of the pleural effusion.

## Discussion

Even in endemic areas, primary pleural coccidioidomycosis is rare. When it has been reported, it is mainly right sided and can be present in all sizes. A pleural fluid lymphocyte-predominant exudative effusion is commonly associated with tuberculosis pleurisy, other fungal infections and lymphoma. In general, exudative pleural effusions present a diagnostic challenge because a wide differential of organisms may potentially cause the effusion, because numerous organs may serve as the foci of infection (Table 1), and because of limitations in commercially available confirmatory studies. This is particularly true when a rare presentation of an infectious organism is observed, as in our case.

**Table 1:** Differential diagnosis for lymphocytic pleural effusion

Tuberculosis
Non-tuberculosis Mycobacterium
Fungal pleurisy
Viral pleurisy
Malignancy
Lymphoma
Solid tumors
Sarcoidosis
Chylothorax
Post-Coronary Bypass Graft
Yellow-Nail Syndrome

The incidence of coccidioidomycosis is increasing, with the majority of reports from the states of Arizona and California. There is particular risk associated with outdoor activity owing to seasonal precipitants and aerosolization of fine sand and silt, particularly in Filipinos and African-Americans.

A number of methods assist in the diagnosis of symptomatic coccidioidomycosis infections, but recognition of its existence is of primary importance. A positive skin reaction can occur as early as 6 to 48 hours after injection, but a positive test is not entirely diagnostic of an active infection; it merely raises suspicion of cellular immunity [4]. The limitations of this test include false-positive test results in individuals vaccinated against or previously exposed to coccidioides. In addition, cross-reactivity with histoplasma capsulatum can occur. The expression of this delayed-type hypersensitivity is lower with disseminated disease.

It is the active and passive immunity that are used in detection and monitoring strategies. Serum immunoglobulin levels are used to detect the presence of an acute infection or immunity, depending on the immunoglobulin type: IgM or IgG. Coccidioides IgM levels may be persistently elevated for up to 6 months in acute infections. Complement fixation is another useful tool for monitoring both the extent of coccidioidomycosis and the response to treatment. Low serum quantified titers of between two and four have been encountered in early-phase coccidioidal infection, limited dissemination and late-stage disease as the titer is declining [5]. A serum complement fixation titer level greater than 32 is generally a sign of disseminated disease. If dissemination is considered, then a lumbar puncture needs to be performed because of the risk of coccidioidomycosis meningitis. Cerebrospinal infection occurs in approximately 35% of patients with disseminated disease, even in the absence of meningeal signs. A cerebrospinal fluid complement fixation titer of 1:2 or greater usually indicates the presence of meningitis [6].

*Coccidioides immitis *can be cultured from tissue and body fluids. Saubolle et al. showed that the respiratory tract has the highest yield of recovery [7]. Cultures may take 3 to 4 weeks to grow, delaying diagnosis. The distinguishing feature is the presence of a thick-walled spherule with endospores. A more rapid approach to identification involves real-time polymerase chain reaction (PCR) [8]. Cross-reactivity comparisons with bacteria, other fungi, mycobacteria and viruses demonstrate 100% specificity to coccidioides. Biopsies and surgical specimen cultures are more likely to result in a positive culture than microscopic examination. Between 25% and 50% of sputum samples, bronchial washings, spinal fluid and urine specimens yield positive cultures [7]. Blood cultures are unlikely to yield the presence of coccidioides, but when positive, they are associated with acute infection, dissemination and a high mortality.

The Infectious Disease Society of America's recommendation for initial therapy of non-meningeal extrapulmonary infection is with an oral azole agent [9]. Clinical trials have shown that fluconazole daily dosage eradicates the disease in a majority of patients. In cases of clinical deterioration, amphotericin B 0.5 to 1.5 mg/kg per day should be administered. The newer extended spectrum azoles voriconazole and posaconazole appear to be effective in small clinical trials but are not yet suitable to be considered as first-line therapy. In small clinical trials, the use of posaconazole in the treatment of refractory coccidioidomycosis shows promising results with minimal side effects [10]. Measuring the response to treatment can be a slow and challenging process. To establish adequate therapy, patients should be routinely followed up every 3 to 6 months for up to 2 years.

## Conclusion

With increased domestic and international travel, coccidioidomycosis will likely be encountered in nonendemic regions. A parapneumonic effusion from pulmonary coccidioidomycosis is seen in up to 50% of cases; primary pleural coccidioidomycosis, however, is a rare clinical feature of an endemic infectious disease. This lymphocytic-predominant effusion mimics other diseases, therefore recognition by physicians is critical for a timely diagnosis and therapy. Tissue culture can assist in the diagnostic approach and PCR analysis shows potential as a possible addition.

## Abbreviations

LDH: lactate dehydrogenase; PCR: polymerase chain reaction.

## Competing interests

The authors declare that they have no competing interests.

## Consent

Written informed consent was obtained from the patient for publication of this case report and accompanying images. A copy of the written consent is available for review by the Editor-in-Chief of this journal.

## References

[B1] Williams FM Markides V Edgeworth J Williams AJ Reactivation of coccidioidomycosis in a fit American visitor Thorax 1998 53 811 812 10319068 10.1136/thx.53.9.811 PMC1745313

[B2] Pinckney L Parker BR Primary coccidioidomycosis in children presenting with massive pleural effusion AJR Am J Roentgenol 1978 130 247 249 414573 10.2214/ajr.130.2.247

[B3] Hamer L Castillo J Coccidioidomycosis presenting as a massive pleural effusion in a postpartum woman Indian J Chest Dis Allied Sci 2006 48 59 62 16482953

[B4] Ampel N Measurement of cellular immunity in human coccidioidomycosis Mycopathologia 2003 156 247 262 10.1023/B:MYCO.0000003580.93839.71 14682448

[B5] Pappagianis D Zimmer B Serology of coccidioidomycosis Clin Microbiol Rev 1990 3 247 268 2200605 10.1128/cmr.3.3.247 PMC358158

[B6] Mandell G Bennet J Dolin R Principles and Practice of Infectious Disease 2004 6 Philadelphia, PA: Elsevier/Churchill Livingstone 3046

[B7] Saubolle MA McKellar PP Sussland D Epidemiologic, clinical and diagnostic aspects of coccidioidomycosis J Clin Microbiol 2007 45 26 30 10.1128/JCM.02230-06 17108067 PMC1828958

[B8] Binnicker MJ Buckwalter SP Eisberner JJ Stewart RA McCullough AE Wohlfiel SL Wegenack NL Detection of coccidioides species in clinical specimen by real-time PCR J Clin Microbiol 2007 45 173 178 10.1128/JCM.01776-06 17108077 PMC1828991

[B9] Cohen J Powderly W Infectious Disease 2004 2 Chicago, IL: Mosby 2371 2372

[B10] Anstead GM Corcoran G Lewis J Berg D Graybill JR Refractory coccidioidomycosis treated with posaconazole Clin Infect Dis 2005 40 1770 1776 10.1086/430303 15909265

